# Auxin and Gibberellins Are Required for the Receptor-Like Kinase ERECTA Regulated Hypocotyl Elongation in Shade Avoidance in Arabidopsis

**DOI:** 10.3389/fpls.2018.00124

**Published:** 2018-02-07

**Authors:** Junbo Du, Hengke Jiang, Xin Sun, Yan Li, Yi Liu, Mengyuan Sun, Zhou Fan, Qiulin Cao, Lingyang Feng, Jing Shang, Kai Shu, Jiang Liu, Feng Yang, Weiguo Liu, Taiwen Yong, Xiaochun Wang, Shu Yuan, Liang Yu, Chunyan Liu, Wenyu Yang

**Affiliations:** ^1^College of Agronomy, Sichuan Agricultural University, Chengdu, China; ^2^Sichuan Engineering Research Center for Crop Strip Intercropping System, Sichuan Agricultural University, Chengdu, China; ^3^Key Laboratory of Crop Ecophysiology and Farming System in Southwest China – Ministry of Agriculture, Sichuan Agricultural University, Chengdu, China; ^4^College of Resources, Sichuan Agricultural University, Chengdu, China

**Keywords:** ERECTA, receptor-like kinase, shade avoidance, auxin, GA

## Abstract

Plants use shade avoidance strategy to escape the canopy shade when grown under natural conditions. Previous studies showed that the Arabidopsis receptor-like kinase ERECTA (ER) is involved in shade avoidance syndrome. However, the mechanisms of ER in modulating SAR by promoting hypocotyl elongation are unknown yet. Here, we report that ER regulated hypocotyl elongation in shade avoidance requires auxin and gibberellins (GAs). The T-DNA insertional *ER* mutant *er-3* shows a less hypocotyl length than that in Col-0 wild type. Promoter::GUS staining analysis shows that *ER* and its paralogous genes *ERECTA-LIKE1* (*ERL1*) and *ERECTA-LIKE2* (*ERL2*) are differentially expressed in the seedlings, of which only *ER* is most obviously upregulated in the hypocotyl by shade treatment. Exogenous feeding assay by using media-application with vertical-grown of Arabidopsis seedlings showed that the hypocotyl length of *er-3* is partially promoted by indol-3-acetic acid (IAA), while it is relatively insensitive of *er-3* to various concentrations of IAA than that of Col-0. Hypocotyl elongation of *er-3* is promoted similar to that of Col-0 by high temperature in the white light condition, but the elongation was not significantly affected by the treatment of the auxin transport inhibitor 1-*N*-naphthylphthalamic acid (NPA). Exogenous GA3 increased the hypocotyl elongation of both *er-3* and the wild type in the shade condition, and the GA3 biosynthesis inhibitor paclobutrazol (PAC) severely inhibits the hypocotyl elongation of Col-0 and *er-3*. Further analysis showed that auxin biosynthesis inhibitors yucasin and L-kynurenine remarkably inhibited the hypocotyl elongation of *er-3* while yucasin shows a more severe inhibition to *er-3* than Col-0. Relative expression of genes regulating auxin homeostasis and signaling, and GA homeostasis is less in *er-3* than that in Col-0. Furthermore, genetic evidences show that *ER* regulated hypocotyl elongation is dependent of PHYTOCHROME B (PHYB). Overall, we propose that ER regulated shade avoidance by promoting hypocotyl elongation is PHYB-dependent and requires auxin and GAs.

## Introduction

Light is one of the most important factors for plant survival and production. In a natural environment, plants always grow closely to one another. Under these conditions, red light wavelengths is been absorbed while the far red light wavelengths is reflected by the leaves of the neighboring plants, resulting in reduction of red:far red (R:FR) light ratio and light intensity, referred to as shade condition. Plants have evolved sophisticated mechanisms which involves architecture and physiological process including increase of the hypocotyl and stem length, hyponasty, early flowering and yield reduction, which is referred to as shade avoidance syndrome (SAR) (Casal, 2013; Wit et al., 2016). In the shade, plants must accelerate their growth in order to maintain their height at least as tall as their neighboring plants to succeed in light sensing competition conditions (Ballaré, 1999). In this process, plants have to expend more energy to support their elongation growth at the expense of leaf development, seed number and yield reduction. Arabidopsis and most crops show typical phenotypes of SAR, while some other plants display shade tolerance phenotype mimicking the phenotypes of the plants in the white light condition (Valladares and Niinemets, 2008; Carriedo et al., 2016).

Upon sensing the canopy shade by the neighboring plants, phytochromes in plant cells would perceive the changes of R:FR light condition and rapidly evoke cascades of actions. Of all the five phytochromes, phytochrome A (PHYA), phytochrome B (PHYB), phytochrome C (PHYC), phytochrome D (PHYD), and phytochrome E (PHYE) in Arabidopsis, PHYB was found to play dominant roles in shade avoidance (Ballaré, 1999; Ruberti et al., 2012; Casal, 2013; Wit et al., 2016). When plants were under shade conditions, PHYB releases the binding of phytochrome interacting factor (PIF) transcription factors and facilitates their entry into the nucleus to bind the promoters of the target genes to trigger the expression of genes in regulating phytohormone levels and signaling pathways (Lorrain et al., 2008; Casal, 2013). Most phytohormones which are found to participate in several aspects of the shade avoidance signaling pathways, auxin and gibberellins (GAs) are best established to be essential for elongation-promoting of plant hypocotyls, stems and petioles (Wit et al., 2016; Yang and Li, 2017). In Arabidopsis and *Brassica rapa* seedlings, auxins are biosynthesized in the cotyledon when suffered to shade and cotyledon-synthesized auxins are then transported to promote the hypocotyl elongation (Tao et al., 2008; Procko et al., 2014). In these processes, genes related to plant growth and development, and adaptation are largely expressed.

Indol-3-acetic acid (IAA) is the predominant naturally occurring auxin in plants (Zhao, 2010, 2012). In higher plants, auxin biosynthesis is likely extremely complex in plants, which includes *de novo* auxin production and the release from auxin conjugates (Zhao, 2010, 2012, 2014). IAA exists in two forms, the free IAA and conjugated IAA, the free IAA can be converted from the conjugated IAA, which is considered as the storage forms or the intermediates for degradation (Woodward and Bartel, 2005; Ludwig-Müller, 2011; Zhao, 2014). Previous isotope-labeling experiment and genetic evidence demonstrated that auxin principally biosynthesized via tryptophan (Trp)-dependent and Trp-independent pathways to coordinately regulate plant growth and development (Wright et al., 1991; Normanly et al., 1993; Woodward and Bartel, 2005; Wang et al., 2015). More evidence showed that several Trp-dependent auxin biosynthesis pathways contribute predominantly to IAA levels referring to the indole-3-acetaldoxime (IAOx) pathway, indoleacetamine (IAM) pathway, and the indole-3-pyruvic acid (IPA) pathway, of which the IPA pathways is the well studied pathway up to date (Korasick et al., 2013; Tivendale et al., 2014; Zhao, 2014). IAA biosynthesized from the Trp by using the IPA as intermediate by a two-step pathway is the best completely established pathway (Zhao, 2012, 2014). In this pathway, Trp is first converted to IPA by TAA1/TARs and IPA is subsequently catalyzed by YUCCAs (YUCs) into IAA (Zhao et al., 2001; Tao et al., 2008). In recent years, more and more compelling evidence showed that, in addition to auxin biosynthesis, auxin transport and metabolism are also essential to hypocotyl elongation in shade avoidance (Pierik et al., 2009; Keuskamp et al., 2010; Zhao, 2010; Yang and Li, 2017). Several studies have demonstrated that auxin transport is important in hypocotyl elongation in etiolated growth, photomorphogenesis, and phototropism similar to shade avoidance response (Jensen et al., 1998; Wu et al., 2016).

When plants are exposed to the adverse environment, the external stimuli will activate the cell membrane-located receptor molecules and initiate the changes of conformation of the receptors. Receptor-like kinases (RLKs) are a set of single transmembrane proteins located on the plasma membrane which involve in sensing the environmental changes including cell-to-cell and cell-to-environment communications (Becraft, 2002; Li, 2010). A typical RLK contains an extracellular domain for signal perception, a transmembrane domain for membrane anchoring and an intercellular Ser/Thr/Tyr kinase domain for signal transduction via phosphorylation (Walker and Zhang, 1990; Shiu and Li, 2004; Oh et al., 2009; Li, 2010; Oh et al., 2010). The first plant RLK was identified from maize by using degenerate PCR primers to the protein kinase domain (Walker and Zhang, 1990). More than 610 RLKs have been found in Arabidopsis in recent years (Shiu and Bleecker, 2001; Shiu et al., 2004). Up to date, more and more RLKs have been found to function in many aspects of plant growth and development, cell death and defense (Li and Chory, 1997; Li et al., 2002; Nam and Li, 2002; Zipfel et al., 2006; Chinchilla et al., 2007; He et al., 2007; Du et al., 2012). For instance, the BRI1 was found as a receptor of brassinosteroids (BRs) (Li and Chory, 1997), BAK1 is a co-receptor of BRI1 in BR signaling pathways in regulating plant growth and development (Li et al., 2002; Nam and Li, 2002). Furthermore, BAK1 was also found as a co-receptor of FLS2 and EFR in pathogen perception and defense pathways (Zipfel et al., 2006; Chinchilla et al., 2007). ERECTA (ER) was firstly found in 1957 by using X-ray irradiated Arabidopsis Landsberg ecotype (Rédei et al., 1992). ER and its functional paralogs ERL1 and ERL2 not only control multiple aspects of plant morphology, but also regulate plant responses to environmental changes (Shpak et al., 2004). Genetic analysis has shown that the *er* mutant displays compact inflorescence and short blunt silique phenotypes due to the decrease in cell proliferation and growth (Shpak et al., 2003). In addition, ERL1 and ERL2 were found to play a redundant role in cell proliferation of organ growth and patterning (Shpak et al., 2004). Furthermore, ER was found to regulate transpiration efficiency in Arabidopsis (Masle et al., 2005). Overexpression of truncated Arabidopsis ER in tomato decreased water loss and enhanced drought tolerance (Villagarcia et al., 2012). Overexpression of Arabidopsis ER in Arabidopsis, rice and tomato increased plant biomass and improved thermal tolerance independent of water content (Shen et al., 2015). Single nucleotide polymorphism (SNP) analysis has shown that an ER homologous gene might be associated with drought adaptation between wild and common bean (Blair et al., 2016).

In recent studies, Patel et al. demonstrated that ER regulates petiole angle and elongation in the shade particularly at cool temperatures in Landsberg ecotype (Patel et al., 2013). It is possible that ER stimulate petiole elongation by promoting the cell expansion in the petioles. Another study also showed that ER makes an important contribution to the shade avoidance syndrome in some stages during plant development against light fluctuations (Kasulin et al., 2013). However, the mechanisms of ER in modulating the SAR are not clear yet, which needs further investigation.

Here, we report our identification of ER in regulating Arabidopsis hypocotyl elongation in the shade in Col-0 background. The results show that loss-of-function of *ER* displays a shorter hypocotyl length than that of Col-0 wild type in the shade condition. Promoter::GUS analyses show that the expression of *ER* is remarkably induced in the hypocotyl by shade treatment. Further investigation show that ER regulated hypocotyl elongation is probably via actions of auxin and GAs, and is dependent of PHYB. Our data supplied a new identification of ER in shade avoidance and detailed a possible mechanism of ER regulated shade avoidance underlying the involvement of auxin homeostasis and signaling pathways, as well as GA homeostasis, which provides new evidence and mechanisms for ER regulated shade avoidance.

## Materials and Methods

### Plant Materials and Growth Conditions

All the Arabidopsis seeds used in these studies were Col-0 ecotype. Seeds of *er-3* (SALK_044110) (Durbak and Tax, 2011), *phyB* (SALK_022035C) were ordered from Arabidopsis Biological Resource Center (ABRC). Plants were grown at 22°C in a long-day growth condition (16 h of light and 8 h of dark) in a greenhouse except those for special treatments.

Arabidopsis seeds were surface-sterilized and grown in the soil or on the 1/2 Murashige and Skoog (MS) media (pH 5.7) supplemented with 1% sucrose and 0.8% agar. For shade treatment of soil-grown plants, 10-day-old Arabidopsis Col-0 and *er-3* single mutant grown in a normal light condition (16 h of light and 8 h of dark) with PAR of 65 μmol m^-2^ s^-1^ and R/FR of 1.3) in a greenhouse and then transferred to a green filter (type No. 122 with a transmittance of 45.6% ^1^, England) with PAR of 31 μmol m^-2^ s^-1^ and R/FR of 0.5 for another 15 days. For shade treatment of media-grown plants, plants were grown in normal light condition for 3 days and then treated with the green filter for another 5 days. Plants were then used for further analyses. Unless it is specially stated that, the plants were grown on slightly vertical 1/2 MS media.

### Semi-quantitative Reverse Transcription (RT)-PCR and Quantitative PCR (qPCR) Analyses

Two micrograms of total RNA were extracted by using an RNAprep pure Plant Kit (Tiangen Biotech) used for reverse transcription with M-MLV (Invitrogen). The first strand of cDNAs was used for semi-quantitative reverse transcription PCR (RT-PCR) analyses with *ExTaq* DNA polymerase (TaKaRa) according to previous studies (Du et al., 2012). Real-time PCR was employed with SYBR^®^ Premix Ex Taq^TM^ II (TaKaRa) and relative expression of genes compared to *ACT2* was calculated using ΔΔCt method. Primers used in this study are listed in **Supplementary Table S1**.

### Promoter::GUS Construction of ER Family Genes and GUS Staining

Promoters of *ER*, *ERL1* and *ERL2* with 1.5 kb were amplified from genomic DNA and cloned with a Gateway^®^ Cloning technology (Invitrogen). The genes were recombined into a binary vector *pBASTA-GWR-GUS* and the destination plasmids were then overexpressed in Col-0 with *Agrobacterium*-mediated transformation. Surface-sterilized seeds of homozygous transgenic plants harboring promoter-GUS were grown on 1/2 MS media. After stratification for 2 days at 4°C, the plates were grown vertically at 22°C in white light for 3 days, and then transferred to white light or shade conditions for another 5 days for GUS staining. For GUS staining, ER, ERL1, or ERL2 promoter-GUS transgenic seedlings were stained according to previous studies (Guo et al., 2010). The stained plants were observed and for photo-capture under a Leica M165C digital stereo microscope, the images were subsequently arranged by using the Adobe Photoshop CS6 software.

### Treatment of Phytohormones and the Biosynthesis Inhibitors

Surface-sterilized seeds were grown on 1/2 MS plates supplemented with 1% sucrose, 0.8% agar and different concentrations of IAA (Sangon), GA3 (Sangon), the auxin biosynthesis inhibitors yucasin and L-kynurenine (kyn) (Sigma-Aldrich), and the GA biosynthesis inhibitor paclobutrazol (PAC) (Sangon). The seedlings were grown at a slight angle with the hypocotyl touching the media. Stock solutions of the phytohormones and the inhibitors were dissolved as follows: 20 mM of IAA were dissolved in ethanol, 20 mM of GA3 in methanol, 500 mM of yucasin in DMSO, 100 mM of kyn in 0.5 M HCl and 5 mM PAC in methanol, respectively. ddH_2_O was used for dilution for the working concentrations. The hypocotyl length was measured with ImageJ 1.6 and analyzed with Graphpad 5.0.

## Results

### Loss-of-Function of *ER* Is Shade Insensitive in Hypocotyl Elongation

**FIGURE 1:**
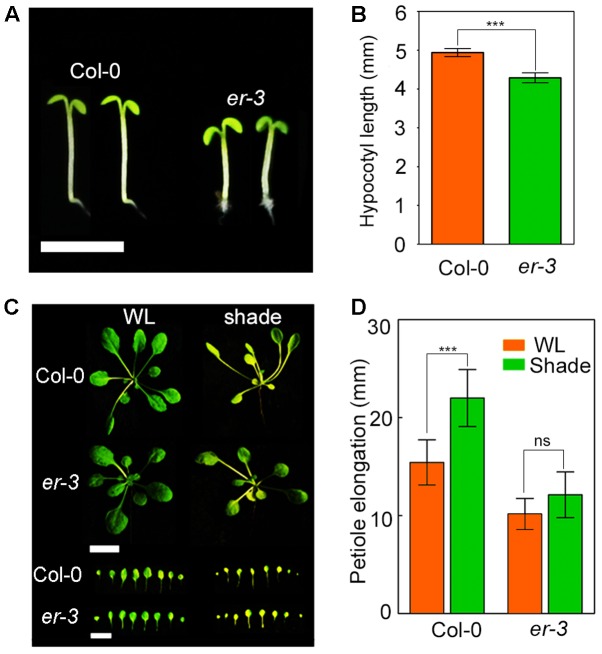
Phenotypes of Col-0 and *er-3* single mutant in shade avoidance. **(A)** Phenotypes of Col-0 and *er-3* grown on MS media under the shade conditions. Scale bar represents 5 mm. **(B)** Statistical data of hypocotyl length of Col-0 and *er-3* under white light and shade conditions. At least 30 seedlings were measured for each genotype. Error bars represent SE. **(C)** Phenotypes of Col-0 and *er-3* grown in the soil under white light and shade conditions. Scale bars represent 10 mm. **(D)** Statistical data of petiole length of Col-0 and *er-3* grown in the soil under white light and shade conditions. At least 30 seedlings were measured for each genotype. Error bars represent SE. Student’s *t*-test indicated the differences are statistically significant (^∗∗∗^*P* < 0.001).

To understand whether ER regulates hypocotyl elongation in shade response, *er-3* (Durbak and Tax, 2011), a T-DNA insertional loss-of-function mutant of *ER*, was used to assess the shade response. After 3-day white-light growth, Col-0 and *er-3* were then moved to the shade condition for another 5 days. The results showed that the hypocotyl length of *er-3* were significantly less sensitive to shade treatment than that of Col-0 in our shade condition (**Figures 1A,B**). To further confirm the shade response phenotypes of *er* mutant, we grew the Col-0 and *er-3* seeds in the soil and covered a green filter for shade treatment. In the shade condition, both soil-grown Col-0 and *er* showed a typical shade avoidance response including petiole elongation (**Figures 1C,D**). Nevertheless, the petiole elongation was shorter in *er-3* single mutant than that in Col-0 in the shade, indicating that petiole elongation of *er-3* is relatively insensitive to shade, which is consistent with other *er* alleles in previous studies (Patel et al., 2013). These results suggest that ER in Col-0 ecotype makes a contribution to shade avoidance syndrome.

### Expression of *AtER* in Hypocotyl Is Upregulated by Shade

To examine whether the transcript level of *ER* in the hypocotyl of Arabidopsis responds to shade, we performed RT-PCR analysis to check gene expression by the time-course shade treatment. The results showed that the shade inducible gene *PIL1* was upregulatedly expressed in the hypocotyls of Col-0 after 30 min by shade treatment and increased as time rises (**Supplementary Figure S1**), indicating that our shade condition is reliable for study on the shade avoidance syndrome. Under this shade condition, we further found that the expression of *ER* was upregulated in the hypocotyls of Col-0 after 30 min and reached at a highest level after 2 h of shade treatment (**Supplementary Figure S1**). These results indicate that *ER* is responsive to shade at the transcription level, suggesting that *ER* might function in shade avoidance pathways in the hypocotyl of Arabidopsis.

### ER Family Genes Are Differentially Responsive to Shade

**FIGURE 2:**
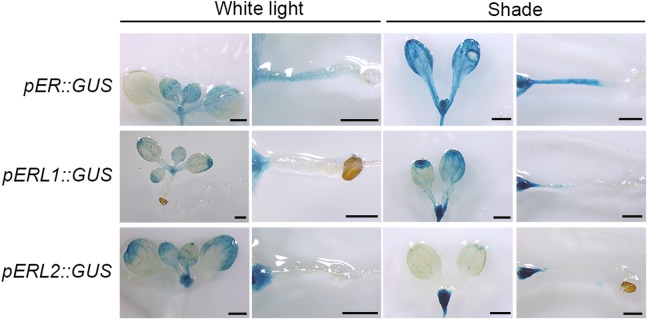
Spatiotemporal expression pattern of *ER* family genes in the white light and shade. Scale bars represent 0.5 mm. pER::GUS staining of 5-day shade treated seedlings show differential expression of *ER*, *ERL1* and *ERL2* under white and shade conditions.

There are three *ER* family genes, *ER* and its paralogous genes *ERL1* and *ERL2* in the Arabidopsis genome. To investigate whether ER family members are responsive to shade stress, we constructed expression vectors harboring a GUS reporter gene driven by the native promoters of *ER* family genes. As shown in **Figure 2**, *ER* is principally expressed in the young tissues and the hypocotyls when grown in the white light, whereas slightly expressed in the cotyledons. However, *ER* shows a higher expression level in the cotyledon, petiole and hypocotyl in the shade condition. *ERL1* is slightly expressed on the leaf margin, petiole and meristem, and its expression is remarkably induced in the meristem by shade but only slightly induced in the hypocotyl. The expression of *ERL2* is mainly distributed on the leaf margin and the meristem of the seedling, whereas downregulated by shade in the leaves and obviously upregulated in the meristem. These results indicate that *ER* family genes differentially respond to shade stress and only the expression of *ER* is most obviously increased in the hypocotyls, suggesting that ER might contribute more to shade avoidance rather than ERL1 and ERL2 in the hypocotyls.

### Exogenous Auxin Promoted the Hypocotyl Length of *er-3* in Shade Response

**FIGURE 3:**
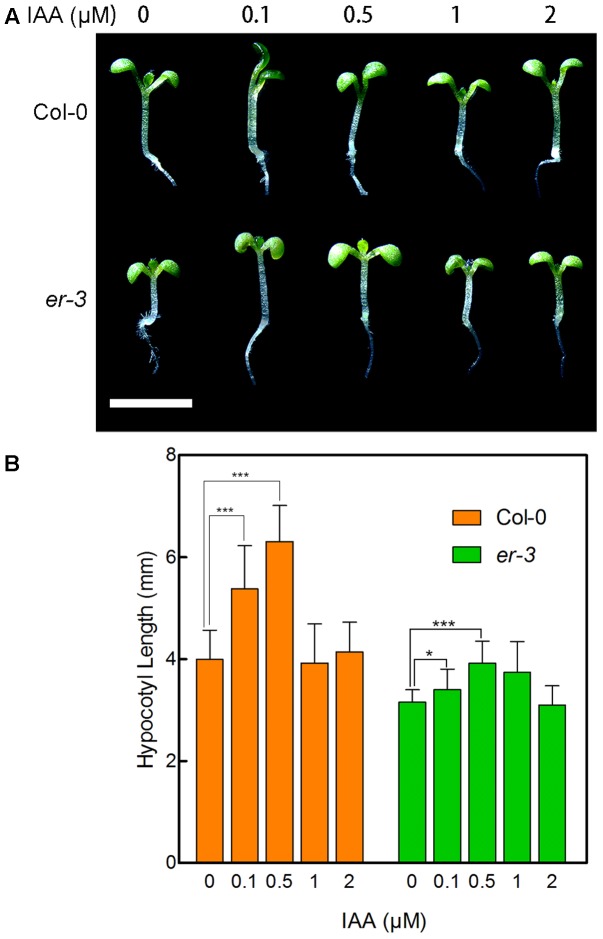
Hypocotyl phenotypes of Col-0 and *er-3* in response to IAA. **(A)** Hypocotyl phenotypes Col-0 and *er-3* seedlings with different concentrations of IAA treatment. **(B)** Statistical data of hypocotyl length of Col-0 and *er-3* by IAA treatment under shade conditions. Student’s *t*-test indicated the differences are statistically significant (^∗^*P* < 0.05; ^∗∗∗^*P* < 0.001). At least 10 seedlings were measured for each genotype. Error bars represent SE. Scale bars represent 0.5 cm.

To examine whether *er-3* inhibited hypocotyl elongation in the shade is impaired in auxin biosynthesis, we determined the response of *er-3* to indole-3-acetic acid (IAA), the main natural auxin. As shown in **Figure 3**, 0.1 and 0.5 μM of IAA remarkably promoted the hypocotyl elongation of both Col-0 and *er-3*, and 0.5 μM of IAA fully rescued the hypocotyl length of *er-3* than that of Col-0 without IAA treatment. The results indicate that IAA is essential for ER regulated hypocotyl elongation and IAA biosynthesis might be impaired in *er-3* in the shade. Further, higher concentrations of IAA inhibit the elongation of both Col-0 and *er-3*, but *er-3* shows a shorter hypocotyl phenotype than that of Col-0 by the same concentration of IAA treatment (**Figures 3A,B**), implying that auxin signaling is also diminished in the *er-3* mutant. These results suggested that both auxin biosynthesis and signaling pathways might be impaired in the *er-3* mutant in the shade condition.

Genetic and physiological evidences show that IAA is principally biosynthesized from the precursor tryptophan via TRYPTOPHAN AMINOTRANSFERASE of ARABIDOPSIS/SHADE AVOIDANCE 3 (TAA1/SAV3), which catalyzes the production of IPA from L-tryptophan (L-Trp), then the IPA is as the substrate of YUC proteins to produce IAA (Zhao et al., 2001; Tao et al., 2008; Dai et al., 2013). Chemical library screening assay showed that kyn is a Trp analog as an effective competitive inhibitor of TAA1/TARs (TRYPTOPHAN AMINOTRANSFERASE RELATEDs) in Arabidopsis, which can effectively block the steps of Trp to IPA (He et al., 2011). In IPA to IAA steps, yucasin was recently found as a potent inhibitor of YUC enzymes in the major IAA biosynthesis pathways (Nishimura et al., 2014). To further understand whether ER regulated shade avoidance is deficient in auxin biosynthesis and in which step of auxin biosynthesis pathways, the effects of kyn and yucasin on Col-0 and *er-3* single mutant were investigated. In the shade condition, the results showed that the hypocotyl elongation of both vertically grown Col-0 and *er-3* at a slight angle are inhibited by both kyn and yucasin treatment (**Supplementary Figures S2A,B**). However, elongation of *er-3* shows less sensitive to kyn and yucasin than that of the wild type (**Supplementary Figures S2A,B**), suggesting that ER regulated hypocotyl elongation might depend on conversion of both Trp to IPA and IPA to IAA. It is reported that in some growth conditions, the plant appears thigmotropic response (Meng et al., 2012). To clarify that whether the inhibition of hypocotyl elongation by kyn and yucasin is resulted from the thigmotropism, we also test the horizontal growth of the plants on agar plates (0.5% agar) feeding by IAA biosynthesis inhibitors in the shade condition. Plants showed a similar inhibition of the hypocotyl elongation of Col-0 and *er-3* by both kyn and yucasin to that grown vertically at a slight angle (**Supplementary Figures S2A,C**). The inhibition of the hypocotyl elongation of Col-0 and *er-3* by IAA biosynthesis inhibitor therefore seems to be independent of thigmotropism. These results indicate that auxin biosynthesis is essential for the hypocotyl elongation of Col-0 and *er-3* stimulated by shading.

### High Temperature Increases Shade-Stimulated Hypocotyl Elongation of *er-3*

Previous studies demonstrated that high temperature can increase the endogenous free auxin levels to promote the hypocotyl elongation (Gray et al., 1998). To test whether *er-3* regulated hypocotyl elongation in the shade is dependent on endogenous auxin levels, we tested the hypocotyl elongation response of *er-3* to a high temperature of 30°C. The results showed that, similar to shade avoidance syndrome, hypocotyl elongation of both Col-0 and *er-3* is enhanced by high temperature even in the white light condition, and hypocotyl length of *er-3* is rescued similar to that of Col-0 by the same high temperature treatment, indicating that endogenous auxin level is essential for ER-modulated hypocotyl elongation. To further investigate that whether auxin transport is also essential for promoting the hypocotyl elongation of *er-3* by endogenous IAA increase, we use a polar auxin transport inhibitor 1-*N*-naphthylphthalamic acid (NPA) treatment at 30°C. As shown in **Supplementary Figure S3**, NPA could slightly inhibit the hypocotyl length of both Col-0 and *er-3* that increased by high temperature in the white light, while the hypocotyl length shows no significance between Col-0 and *er-3* by 5 μM of NPA treatment. These results suggest that ER-mediated hypocotyl elongation is dependent on endogenous auxin levels but not remarkably depends on auxin transport.

### Exogenous GA3 Increased the Hypocotyl Length of *er* in the Shade

**FIGURE 4:**
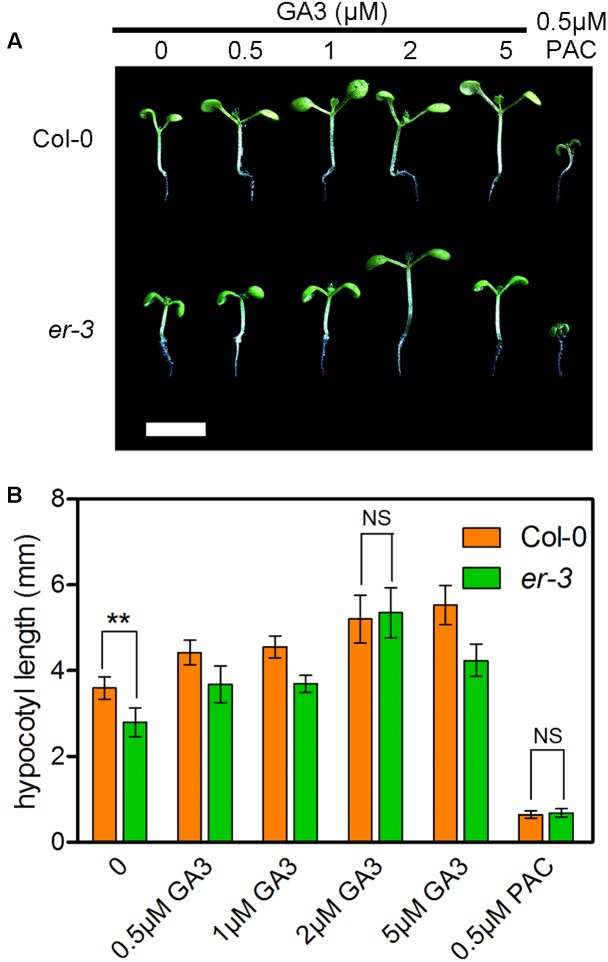
Exogenous feeding of GA3 enhanced the hypocotyl elongation of both Col-0 and *er-3* in the shade. **(A)** Hypocotyl phenotypes of Col-0 and *er-3* by different concentrations of GA3 and PAC treatment. Scale bar represents 0.5 cm. **(B)** Statistical data of hypocotyl length of Col-0 and *er-3* by treatment of GA3 and PAC under shade conditions. Student’s *t*-test indicated the differences are statistically significant (^∗∗^*P* < 0.01). At least 10 seedlings were measured for each genotype. Error bars represent SE.

Gibberellins are another important group of phytohormones required to regulate the hypocotyl elongation in shade avoidance. To understand whether ER modulated hypocotyl elongation is deficient in GA biosynthesis, the effects of various concentrations of GA3 on the hypocotyl elongation of *er-3* and Col-0 were investigated (**Figure 4**). The results showed that lower concentrations of GA3 can promote the hypocotyl elongation of both Col-0 and *er-3*, and the promotion of the hypocotyl length in *er-3* is less than that in Col-0 by concentrations of 0.5 and 1 μM of GA3 (**Figures 4A,B**). However, 2 μM of GA3 can stimulate the hypocotyl elongation of *er-3* similar to that of Col-0 (**Figures 4A,B**), indicating that the ER regulated hypocotyl elongation is partially dependent of GAs. Furthermore, the hypocotyl elongation of both Col-0 and *er-3* are impaired to the similar length by the GA biosynthesis inhibitor PAC (**Figures 4A,B**), indicating that GAs are important, but not only specific, for ER-mediated shade avoidance. These results suggest that GA level is an important regulator for hypocotyl growth for both the wild type and *er-3* mutant in the shade.

### Auxin- and GA-Related Genes Are Differentially Regulated by ER in the Shade

To further elucidate the molecular mechanisms of ER modulated hypocotyl growth, genes related to auxin and GA biosynthesis and signaling pathways were examinated. Auxin biosynthesis gene *YUC9* and auxin-responsive genes, *IAA29* and *SAUR68*, whose expression is also rapidly upregulated by shade (Tao et al., 2008; Galstyan et al., 2011), are upregulated in both Col-0 and *er-3* under shade condition, and that the expression level is higher in Col-0 than that in *er-3*, suggesting that *ER* mutation impairs the expression level of auxin biosynthesis gene *YUC9* and auxin response might be impaired in *er-3* in the shade. Previous studies revealed that *VAS2*, encoding an IAA-amido synthetase Gretchen Hagen 3 (GH3).17, is expressed predominantly in the hypocotyl and plays important role in conversion of free IAA to IAA-Glu (IAA-glutamate) independent of IPA-mediated IAA biosynthesis (Zheng et al., 2016). To test whether ER regulated hypocotyl elongation in the shade is dependent on *VAS2*, we checked the expression level of *VAS2* in Col-0 and *er-3*. The results showed that relative expression of *VAS2* is decreased in Col-0 by shading compared to that in the white light condition. While *VAS2* is significantly upregulated in shade-treated *er-3* compared to that in *er-3* grown in the white light, and is much higher in expression than that of Col-0 in the shade condition. These results indicate that *VAS2* might also contribute to ER-regulated hypocotyl elongation in the shade.

**FIGURE 5:**
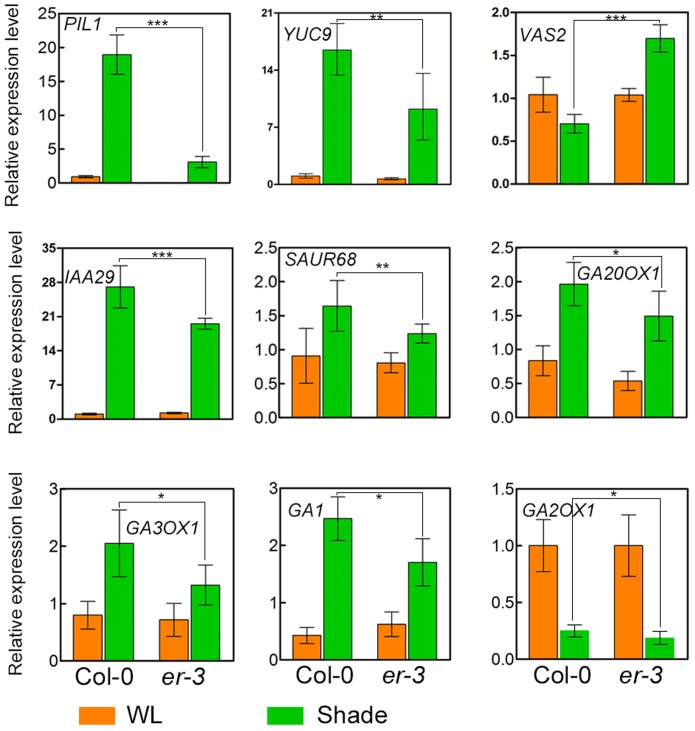
Relative expression of genes in Col-0 and *er-3* under white light and shade conditions. Expression of the shade-inducible gene *PIL1*, auxin biosynthesis gene *YUC9*, auxin homeostasis gene *VAS2*, auxin signaling gene *IAA29* and *SAUR68*, GA biosynthesis gene *GA20OX1*, *GA3OX1* and *GA1* and GA catabolism gene *GA2OX1* were examined by using real time PCR analysis. Similar results from two biological repeats were obtained and at least three technical repeats in each biological assay were performed for the gene expression analysis and a presentative one is shown. Student’s *t*-test indicated the differences are statistically significant (^∗^*P* < 0.05; ^∗∗^*P* < 0.01; ^∗∗∗^*P* < 0.001).

The GA biosynthetic genes *GA20OX1*, *GA3OX1*, and *GA1* were induced in the shade treated both Col-0 and *er-3*, respectively. While expression of *GA3OX1* is less in *er-3* than that in Col-0, indicating that GA biosynthesis might be diminished in the conversion of GA9/GA20 to GA4/GA1. Expression of *GA1* is induced in both Col-0 and *er-3* by shade, whereas the expression level is lower in *er-3* than that in Col-0 (**Figure 5**). However, the expression of the GA catabolic gene, *GA2OX1* is sharply decreased in both shade treated Col-0 and *er-3* than that in white light-treated plants. *GA2OX1* exhibit a slightly less expression in shade treated *er-3* than that in shade-treated Col-0, suggesting that conversion of copalyl diphosphate (CPP) from geranylgeranyl diphosphate (GGPP) in GA biosynthesis might be impaired in *er-3*. However, significant changes in expression of GA biosynthesis and catabolic genes were not more remarkable than that of genes in auxin homeostasis and signaling pathways, implying that auxin might contribute more to ER-regulated hypocotyl elongation than GA in the shade.

### ER Regulated Hypocotyl Elongation in Response to Shade Is Dependent of PHYB

**FIGURE 6:**
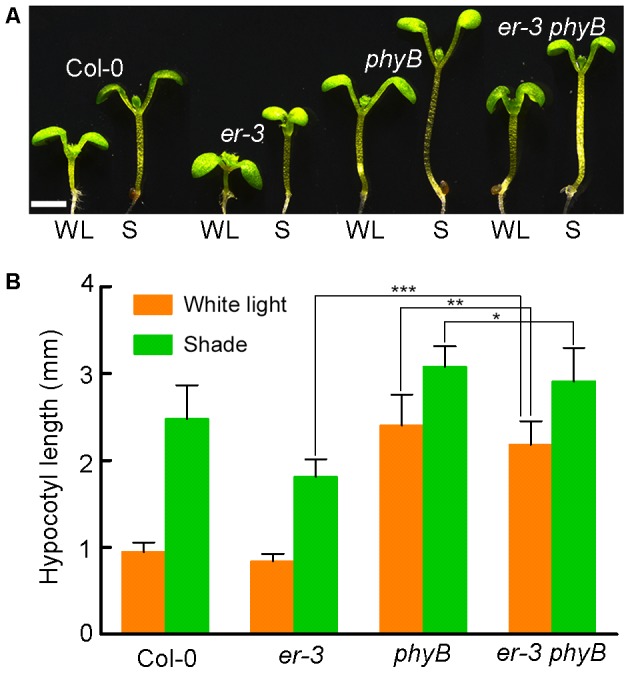
ER promoted hypocotyl elongation is dependent of PHYB in the shade. **(A)** Phenotypes of Col-0, single and double mutants of *er-3* and *phyB* grown in white light and shade conditions. Scale bar represents 1 mm. **(B)** Statistical data of hypocotyl length of Col-0, *er-3* and *phyB* mutants in white light and shade conditions. Student’s *t*-test indicated the differences are statistically significant (^∗^*P* < 0.05; ^∗∗^*P* < 0.01; ^∗∗∗^*P* < 0.001). At least 10 seedlings were measured for each genotype. Error bars represent SE.

Pants use red and far-red light-absorbing phytochromes A and B to sense the changes of R:FR ratio, of which phytochrome B (PHYB) play dominant role in shade avoidance inhibition. To determine whether ER-controlled hypocotyl elongation is via PHYB, we crossed *er-3* with a T-DNA insertional mutant *phyB* to generate an *er-3 phyB* double mutant (**Figure 6A**). In the white light condition, Col-0 and *er-3* single mutant show a hypocotyl inhibition phenotype, and *phyB* single mutant shows a hypocotyl elongation phenotype compared with that of Col-0 and *er-3* single mutant. However, *er-3 phyB* double mutant displays a hypocotyl elongation phenotype mimicking *phyB* single mutant but *ER* mutation significantly reduced hypocotyl elongation of *phyB* (**Figures 6A,B**). These results indicate that PHYB inhibited hypocotyl elongation in the white light is ER dependent. In the shade condition, *er-3* single mutant shows a shade avoidance phenotype in the hypocotyl, but elongation of the hypocotyl of *er-3* was significantly insensitive to shade compared with Col-0. Hypocotyls of both *phyB* and *er phyB* mutants also elongated in the shade condition. However, *er-3 phyB* is less sensitive to shade compared to the *phyB* single mutant, but *er-3 phyB* double mutant shows more sensitive in hypocotyl elongation compared to that of *er* single mutant, indicating that ER promoted hypocotyl elongation depends on PHYB (**Figures 6A,B**).

## Discussion

Hypocotyl growth is stimulated by various factors from internal signals and surrounding environment and controlled by complicated signaling networks. Plant receptor-like kinases play critical roles in perception of environmental signals. However, researches on RLK-mediated shade avoidance are seldom reported. Previous studies demonstrated that the leucine-rich repeat receptor-like kinase ER not only regulates Arabidopsis growth and development, but also plays a role in response to environmental stimuli (Torii et al., 1996; van Zanten et al., 2009; Shpak, 2013). Our study demonstrated that a T-DNA insertional mutation of Arabidopsis *ER* in Clo-0 background inhibits hypocotyl elongation in shade avoidance. Previous studies showed that Arabidopsis ER regulates petiole elongation and leaf hyponasty response in shade avoidance in a temperature-dependent manner in the Arabidopsis Landsberg ecotype (Patel et al., 2013). Another study from an independent group showed that ER contributes to hypocotyl, petiole and lamina elongation, hyponastic growth and flowering time in response to end-of-day far-red (R/FR) light in a genetic background-dependent manner (Kasulin et al., 2013). However, mechanisms of ER controlled shade avoidance are unknown yet.

Our physiological evidence revealed that ER regulates hypocotyl elongation in shade avoidance probably via auxin homeostasis and signaling pathways, and GA homeostasis pathways. Firstly, expression pattern analyses showed the expression of *ER*, but not its homologs *ERL1* and *ERL2*, which is specifically induced by shade in the Arabidopsis hypocotyl, suggesting that ER might play roles in hypocotyls in shade avoidance distinct from ERL1 and ERL2. Moreover, a cis-acting regulatory element prediction of the ER promoter region by PlantCARE showed that 14 light responsive elements are found in the promoter of *ER* (**Supplementary Figure S4**), providing a clue that ER might involve in light responsiveness. Secondly, exogenous IAA feeding promoted the hypocotyl elongation of both Col-0 and *er-3* in the shade, while exogenous IAA only partially promoted the hypocotyl elongation of *er-3* comparing to that of the wild type, suggesting that ER-mediated hypocotyl elongation might be partially dependent of auxin biosynthesis. Nevertheless, *er-3* was relatively insensitive to IAA treatment than the wild type in the same concentration of IAA in the shade (**Figure 3**), implying that the auxin signaling pathways might be also impaired in the *er-3* mutant compared to the wild type in the shade. Previous studies showed that indole-3-pyruvic acid (IPyA) pathway is a major IAA biosynthesis pathway from tryptophan in Arabidopsis, in which the tryptophan aminotransferase SAV3/TAA1 and YUC enzymes play principal roles (Zhao et al., 2001; Tao et al., 2008; Dai et al., 2013). By using a chemical biology approach, Nishimura et al. discovered a potent IAA biosynthesis inhibitor, 5-(4-chlorophenyl)-4H-1,2,4-triazole-3-thiol (yucasin), which can effectively inhibit the activities of YUC thus impaired IAA production *in planta* (Nishimura et al., 2014). Another study showed that kyn is a tryptophan analog which can effectively inhibit Arabidopsis SAV3/TAA1/TARs identified from a chemical library screen (He et al., 2011). Our results showed that both yucasin and kyn can effectively inhibit the hypocotyl elongation of Col-0 and *er-3*, suggesting that both yucasin and kyn are potent inhibitors for Arabidopsis in the shade. Thirdly, it is known that high temperature can increase the endogenous auxin levels to promote the hypocotyl elongation of Arabidopsis and the expression of auxin biosynthesis gene *YUC9* is also induced by shade (Gray et al., 1998; Müller-Moulé et al., 2016), our results revealed that high temperature can enhance the hypocotyl elongation of *er-3* similar to that of Col-0 in the white light condition (**Supplementary Figure S3**) and auxin biosynthesis gene *YUC9* is less induced by shade in *er-3* than that in Col-0 (**Figure 5**), indicating that endogenous auxin homeostasis makes an important contribution to ER-mediated hypocotyl elongation in the shade. This conclusion also supported by another recent study which revealed that auxin biosynthesis is essential for ER regulated cell elongation in the hypocotyl under white light (Qu et al., 2017). It is reported that VAS2 controls the endogenous free IAA levels by conversion from IAA-Glu in the hypocotyl epidermis to promote the hypocotyl elongation (Zheng et al., 2016). Our qPCR results showed that relative expression of *VAS2* is down-regulated in Col-0 but upregulated in *er-3* by shading (**Figure 5**), suggesting that auxin metabolism might also contribute to ER-mediate shade avoidance in hypocotyl elongation. Auxin transport also plays roles in plant hypocotyl elongation in the white light condition (Jensen et al., 1998; Wu et al., 2016), our results indicated that auxin transport might be not essential for ER-regulated hypocotyl elongation, in that NPA treatment cannot cause the significant reduce in hypocotyl elongation of *er-3* compared to that of Col-0 in white light with high temperature conditions. These results suggested that auxin transport seems to be less important than auxin homeostasis in ER-mediated hypocotyl elongation in shade avoidance.

To date, the mechanisms of actions for auxin transport inhibitors have remained poorly understood (Teale and Palme, 2017). Although our physiological assays suggested that auxin transport inhibitors did not directly significantly affect the hypocotyl growth of *er-3* than that of Col-0, previous studies reported that long-term application of high concentrations of auxin to the roots leads to changes of auxin transport probably through auxin-dependent transcriptional control (Sieberer et al., 2000; Vieten et al., 2005; Vanneste and Friml, 2009). Moreover, high temperature is able to impact on membrane fluidity vesicular trafficking, and other hormonal responses (Kim and Portis, 2005; Asensi-Fabado et al., 2013; Hanzawa et al., 2013). For this reason, we speculate that it is also probably that high temperature induced auxin maxima in the roots or other processes and thus resulted in changes of auxin transport. In addition, several evidences has revealed that NPA probably binds either directly to the auxin efflux carrier including PIN and ABC-transporters (Rubery, 1979; Sussman and Goldsmith, 1981) or to auxin efflux-related regulatory proteins and cytoskeleton (Cox and Muday, 1994; Bailly et al., 2008) to inhibit auxin transport, but it has also been proposed that NPA may have other effects independent of auxin transport (Hössel et al., 2005). Altogether, we could not rule out that auxin balance in the hypocotyl of *er-3* might be neither affected by a loss or accentuation of an auxin maxima in the root, leading to alteration of auxin transport, nor affected by other biological processes, which might finally result in changes of ER-mediated hypocotyl elongation. In the future, more molecular and physiological evidences are required to uncover whether and how auxin transportation involving auxin transporters contributes in ER-regulated hypocotyl growth in shade avoidance.

Our exogenous GA3 feeding increased the hypocotyl length of *er-3* to the length of the wild type in the shade, suggesting that GA3 biosynthesis might be diminished in *er-3* under shade condition. Relative expression of genes in GA biosynthesis and catabolism pathways in *er-3* are less than that in the wild type, suggesting that ER modulates hypocotyl elongation might depend on GA homeostasis. Nevertheless, more genetic and biochemical evidences need to be done to elucidate the detailed mechanisms underlying the crosstalk between auxin and GA in ER regulated shade avoidance in the future. PHYB is the main photoreceptor in perception of changes of light quality of red to far-red light wave length. Previous studies show that ER modulates low light intensity induced petiole elongation independent of PHYB (van Zanten et al., 2010). Our genetic evidence shows that loss-of-function of *PHYB* can promote the hypocotyl elongation of *er-3*, whereas the hypocotyl length of the double mutant of *er-3 phyB* is shorter than that of *phyB* single mutant, suggesting that ER regulated hypocotyl elongation depends on PHYB, and *phyB*-mediated promotion of hypocotyl elongation is partially dependent of ER. However, the detailed molecular mechanisms of how does PHYB function in ER-mediated shade avoidance pathways needs further investigation. Interestingly, protein–protein interaction analyses by Search Tool for the Retrieval of Interacting Genes/Proteins (STRING^2^) show that ER and PHYB are in the same protein complex (**Supplementary Figure S5**), thus we speculate that it is probably ER and PHYB function together in hypocotyl elongation in a same protein complex. Furthermore, extensive evidence showed that RLKs play roles in plant growth and development, and defense with their co-receptors as receptor complex in previous studies. For instance, the BR receptor BRASSINOSTEROID INSENSITIVE 1 (BRI1) interacts with its co-receptors SOMATIC EMBRYOGENESIS RECEPTOR KINASEs (SERKs) to perceive and transduce BR signals to regulate plant growth and development (Li et al., 2002; Nam and Li, 2002; Gou et al., 2012). EF-TU RECEPTOR (EFR) and FLAGELLIN-SENSING 2 (FLS2) interacts with SERKs to sense the flagellin22 in plant defense signaling pathways (Zipfel et al., 2006; Chinchilla et al., 2007; Roux et al., 2011; Stegmann et al., 2017). ER family receptors interact with a receptor-like protein TOO MANY MOUTH (TMM) to form a homo- and heterodimer receptor complex to regulate stomatal development (Shpak et al., 2005). Receptor-like kinases FERONIA and THESEUS1 were found to control the shoot elongation of *Geranium pyrenaicum* identified by transcriptome analyses (Gommers et al., 2017). We therefore hypothesize that ER may function with unknown co-receptors to regulate hypocotyl elongation in shade avoidance by regulating the auxin homeostasis and signaling and GA homeostasis related networks. Further studies will focus on disclosing the detailed molecular mechanisms, including the crosstalk of auxin and GA in ER regulated hypocotyl elongation in shade avoidance.

## Author Contributions

JD and WY designed the experiments. JD, HJ, XS, YaL, MS, YiL, ZF, QC, LF, JS, KS, JL, WL, FY, TY, XW, SY, LY, and CL performed the experiments. JD, HJ, XS, and YaL analyzed the data. JD, HJ, XS, and YaL wrote the manuscript.

## Conflict of Interest Statement

The authors declare that the research was conducted in the absence of any commercial or financial relationships that could be construed as a potential conflict of interest.

## Footnotes

1 www.q-max.net

2 http://string-db.org

## Supplementary Material

FIGURE S1 Time course of gene expression in Col-0 induced by shade. *ER* and the shade inducible genes *PIL1* and *XTR7* were upregulated in the shade. *ACT2* was amplified for 23 cycles as an internal control.

FIGURE S2 Exogenous feeding of kyn and yucasin can inhibit shade stimulated hypocotyl elongation. **(A)** Phenotypes of Col-0 and *er-3* seedlings vertically with a slight angle or horizontally grown with treatment of different concentrations of kyn and yucasin in the shade. Scale bar represents 1 cm. **(B)** Statistical data of hypocotyl length of vertically grown Col-0 and *er-3* with a slight angle treated by kyn and yucasin under shade condition. **(C)** Statistical data of hypocotyl length of horizontally grown Col-0 and *er-3* treated by kyn and yucasin under shade condition. Student’s *t*-test indicated the differences are statistically significant (^∗∗∗^*P* < 0.001). At least 15 seedlings were measured for each genotype. Error bars represent SE.

FIGURE S3 High temperature promoted hypocotyl elongation of both Col-0 and *er-3*. **(A)** Phenotype of Col-0 and *er-3* treated with 22°C and 30°C in the while light or shade conditions. 5 μM of NPA feeding were used at 30°C in the shade. **(B)** Statistical data for Col-0 *er-3* with/without NPA treatment grown at 22°C and 30°C in the while light or shade conditions.

FIGURE S4 Motif prediction of the promoter of *ER* by PlantCARE (http://bioinformatics.psb.ugent.be/webtools/plantcare/html/). The yellow boxes show the motifs are light responsive elements.

FIGURE S5 Prediction of interacting proteins of PHYB and ER in Arabidopsis by STRING (http://string-db.org/).

TABLE S1 Primers used in this study.
