# Supplementary figures and images for: Auxin and Gibberellins Are Required for the Receptor-Like Kinase ERECTA Regulated Hypocotyl Elongation in Shade Avoidance in Arabidopsis
Source: Front Plant Sci. 2018 Feb 7;9:124. doi: 10.3389/fpls.2018.00124 (PMC5808342; doi:10.3389/fpls.2018.00124)

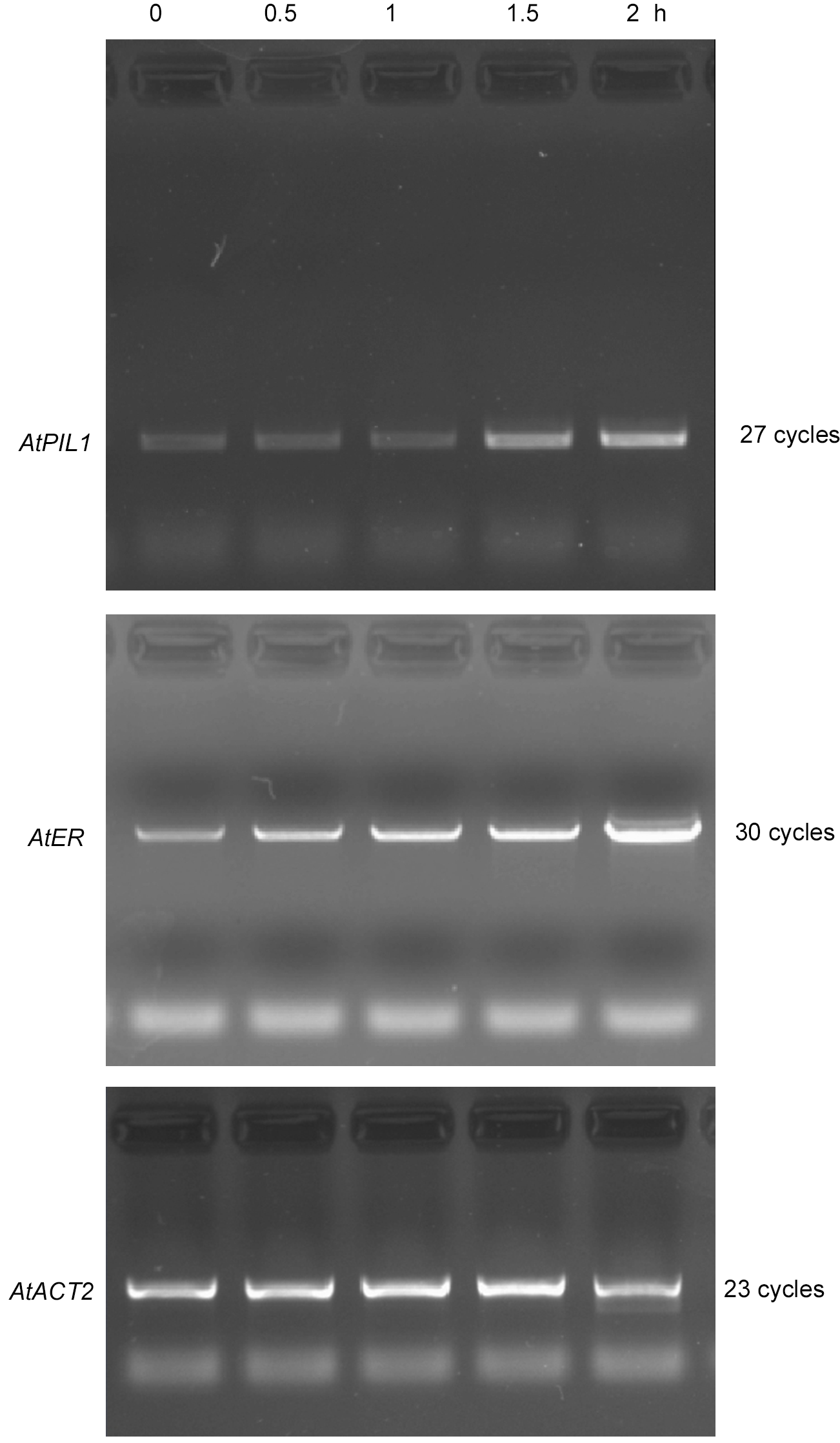

Supplement: FIGURE S1 — Time course of gene expression in Col-0 induced by shade. ER and the shade inducible genes PIL1 and XTR7 were upregulated in the shade. ACT2 was amplified for 23 cycles as an internal control. [file Image_1.TIF]

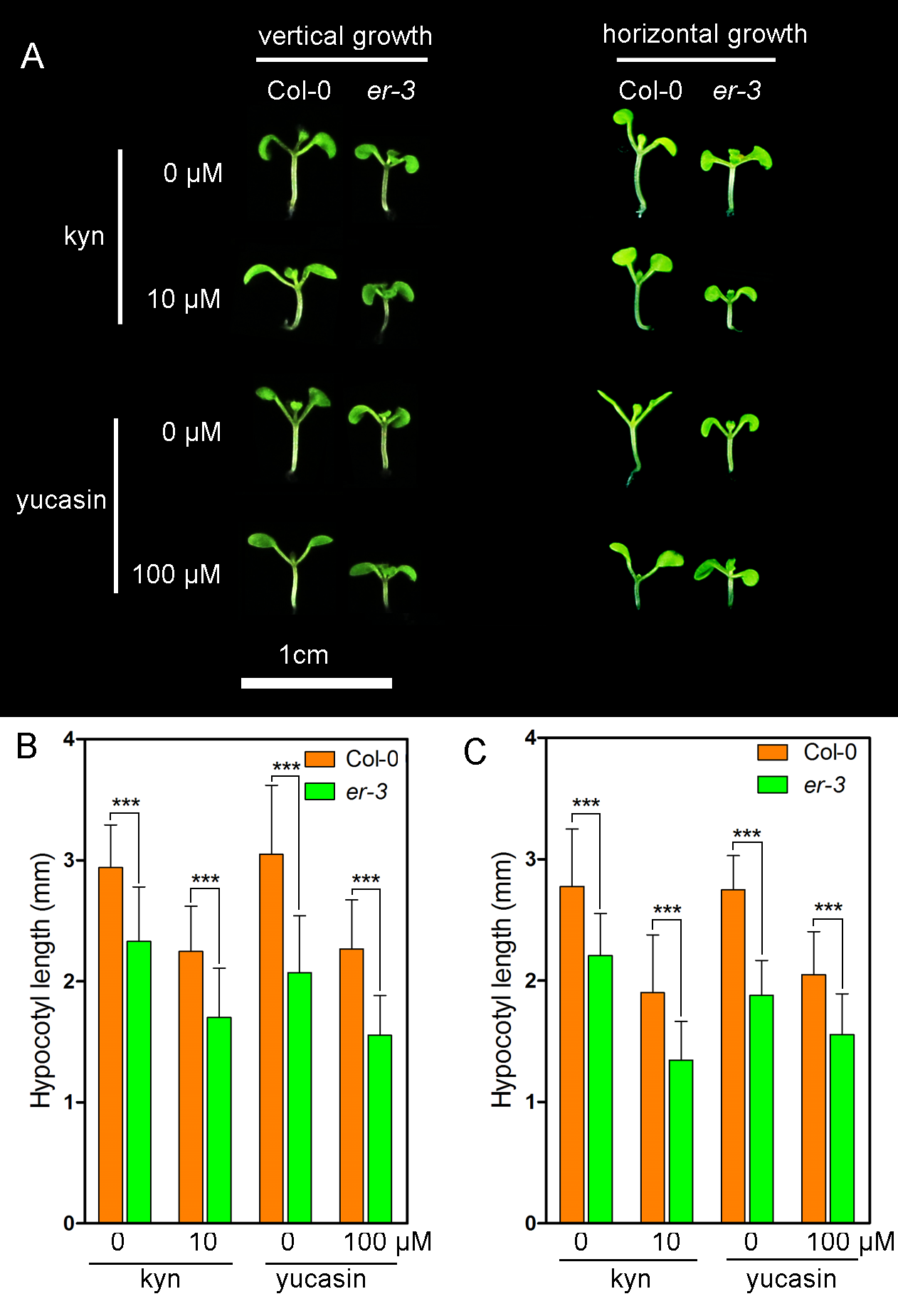

Supplement: FIGURE S2 — Exogenous feeding of kyn and yucasin can inhibit shade stimulated hypocotyl elongation. (A) Phenotypes of Col-0 and er-3 seedlings vertically with a slight angle or horizontally grown with treatment of different concentrations of kyn and yucasin in the shade. Scale bar represents 1 cm. (B) Statistical data of hypocotyl length of vertically grown Col-0 and er-3 with a slight angle treated by kyn and yucasin under shade condition. (C) Statistical data of hypocotyl length of horizontally grown Col-0 and er-3 treated by kyn and yucasin under shade condition. Student’s t-test indicated the differences are statistically significant (∗∗∗P < 0.001). At least 15 seedlings were measured for each genotype. Error bars represent SE. [file Image_2.TIF]

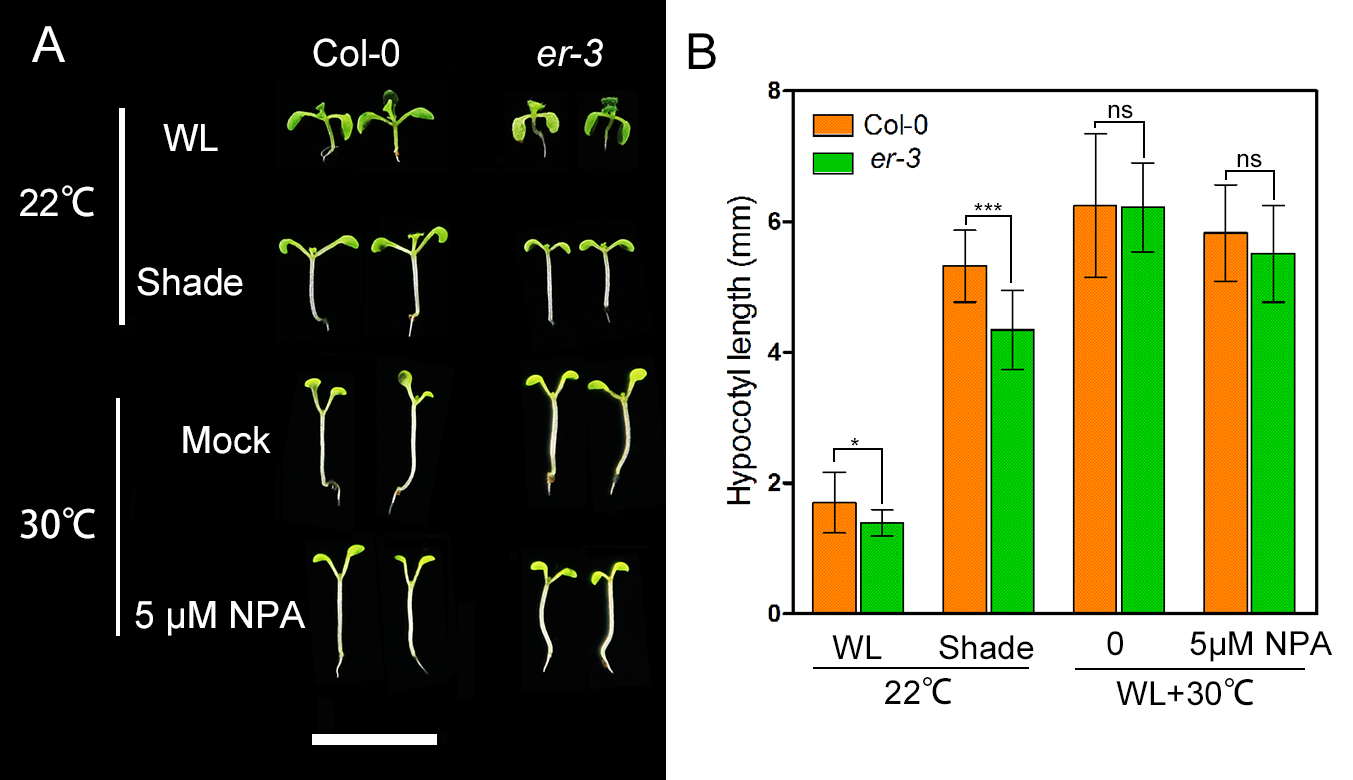

Supplement: FIGURE S3 — High temperature promoted hypocotyl elongation of both Col-0 and er-3. (A) Phenotype of Col-0 and er-3 treated with 22°C and 30°C in the while light or shade conditions. 5 μM of NPA feeding were used at 30°C in the shade. (B) Statistical data for Col-0 er-3 with/without NPA treatment grown at 22°C and 30°C in the while light or shade conditions. [file Image_3.TIF]

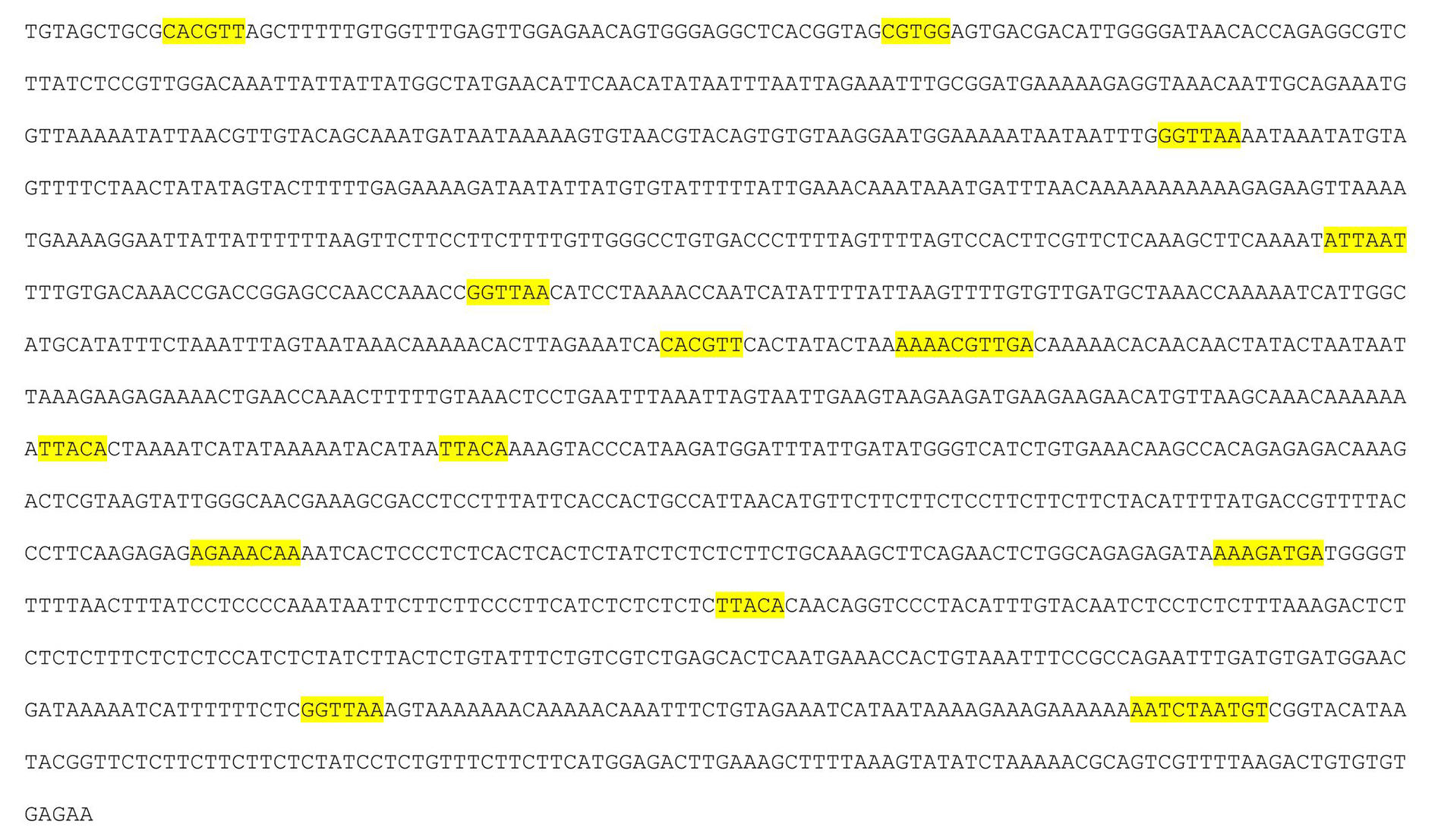

Supplement: FIGURE S4 — Motif prediction of the promoter of ER by PlantCARE (http://bioinformatics.psb.ugent.be/webtools/plantcare/html/). The yellow boxes show the motifs are light responsive elements. [file Image_4.TIF]

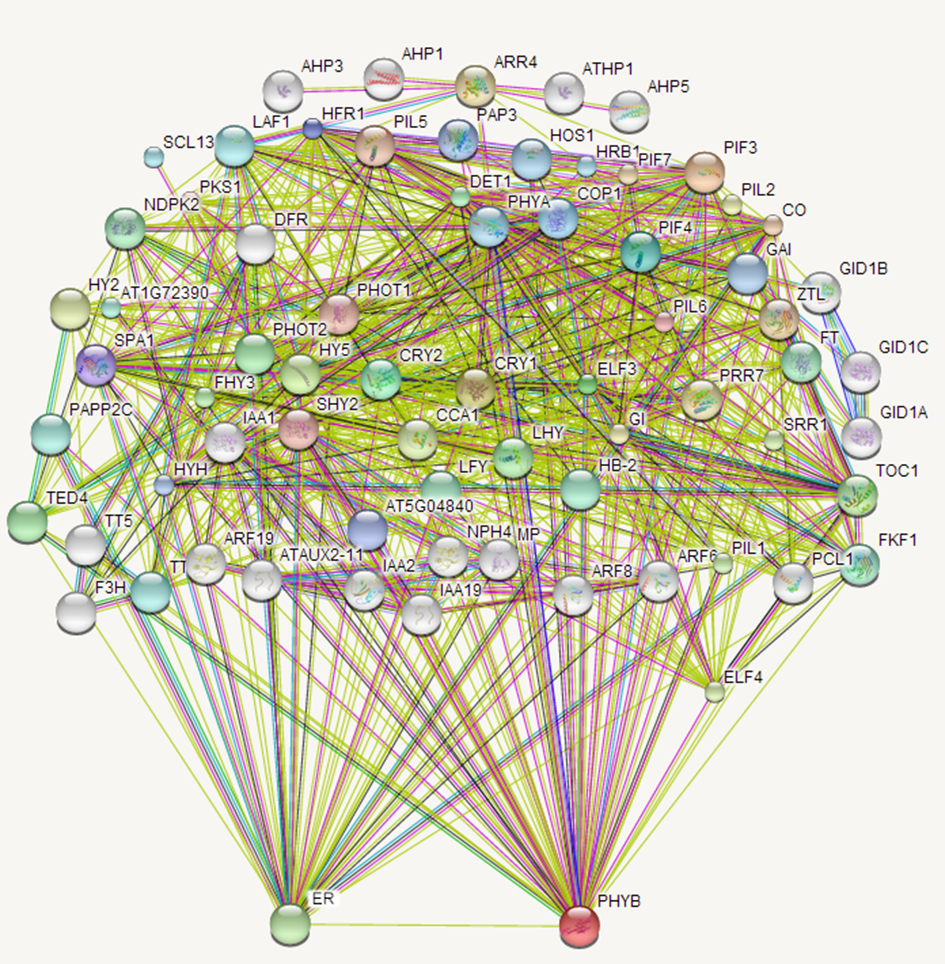

Supplement: FIGURE S5 — Prediction of interacting proteins of PHYB and ER in Arabidopsis by STRING (http://string-db.org/). [file Image_5.TIF]
