# Supplementary material for: Auxin and Gibberellins Are Required for the Receptor-Like Kinase ERECTA Regulated Hypocotyl Elongation in Shade Avoidance in Arabidopsis
Source: Front Plant Sci. 2018 Feb 7;9:124. doi: 10.3389/fpls.2018.00124 (PMC5808342; doi:10.3389/fpls.2018.00124)
Supplement: TABLE S1 — Primers used in this study. [file Table_1.DOCX]

**Table S1. Primers used in this study.**

| **Primer name** | **Primer sequences (5' to 3')** | **Note** |
| --- | --- | --- |
| ACTIN2-F | AGCGCTGAGGCTGATGATATTCAAC | Primers used for semi-quantitative PCR. |
| ACTIN2-R | TCTAGAAACATTTTCTGTGAACGATTC |  |
| ER-F | AAAAAGCAGGCTTCATGGCTCTGTTTAGAGATATTGTTC |  |
| ER-R | AGAAAGCTGGGTCCTCACTGTTCTGAGAAATAACTTGTC |  |
| PIL1-F | ATGCTGCTCTTCATTAACCC |  |
| PIL1-R | TTATGGACTTCTGTGCTTCG |  |
| PIL1q-F | GTGTTTCTCAGACTCAGGCTACTTC | Primers used for qPCR. |
| PIL1q-R | CGGACGCAGACTTTGGGAATTG |  |
| AtYUC9-qF | TCTCTTGATCTTGCTAACCACAATGC |  |
| AtYUC9-qR | CCACTTCATCATCATCACTGAGATTCC |  |
| AtVAS2-qF | ACGCAGACACGTCATCAATCCC |  |
| AtVAS2-qR | TGCTGTGACGTGGCTTTAGCTC |  |
| AtIAA29-qF | CGAGGGTGCTGCGTCTTGTTTG |  |
| AtIAA29-qR | CACGATGATGATACGGGCAATGATGG |  |
| SAUR68q-F | GAGCAACTCTATTGTCCAA |  |
| SAUR68q-R | TCTCCATCCATTCGTCTT |  |
| GA20OX1q-F | GCAGATTCTCCACTAAGC |  |
| GA20OX1q-R | AACTCTTGTCCTAATGTATCG |  |
| GA3OX1q-F | GGTATAGAGGCGATTCAAC |  |
| GA3OX1q-R | TCTTCCACATCCTATCCAA |  |
| GA2OX1q-F | CGTTGAAGAAGAAGGAAGT |  |
| GA2OX1q-R | TGGTAAGAATGTGATTGGTT |  |
| GA1q-F | AGCGTCATGAAACGGTTGAGTCAGTG |  |
| GA1q-R | TGCCAACCCAACATGAGACAGC |  |
| ACTIN8-F | GTGTCTGGATTGGTGGTTCTA |  |
| ACTIN8-R | TGCTTCATCATACTCTGCCTTA |  |
